# Junk food consumption and socio-demographic correlates among health sciences students in the UAE

**DOI:** 10.3389/fnut.2026.1742473

**Published:** 2026-02-12

**Authors:** Vimala Edwin, Mohamedanas Mohamedfaruk Patni, Ramya Kundayi Ravi, Priyalatha Muthu, Sirajunisa Talath, Nallan C. S. K. Chaitanya, Veronica Edwin Nayagam

**Affiliations:** 1 RAK College of Nursing, RAK Medical and Health Sciences University, Ras Al Khaimah, United Arab Emirates; 2 RAK College of Medical Sciences, RAK Medical and Health Sciences University, Ras Al Khaimah, United Arab Emirates; 3 RAK College of Pharmacy, RAK Medical and Health Sciences University, Ras Al Khaimah, United Arab Emirates; 4 RAK College of Dental Sciences, RAK Medical and Health Sciences University, Ras Al Khaimah, United Arab Emirates; 5 RAK Medical and Health Sciences University, Ras Al Khaimah, United Arab Emirates

**Keywords:** body mass index, eating habits, energy drinks, food habits, students

## Abstract

**Background/objectives:**

Junk food consumption is rising among university students despite well-documented health risks. This study examines the socio-demographic factors influencing junk food consumption among health professional students in the UAE.

**Methods:**

A validated, semi-structured questionnaire was used to assess junk-food awareness, consumption patterns, and behavioral preferences among health-professional students. The tool comprised sections on sociodemographic characteristics, awareness of junk food as unhealthy, consumption frequency, portion size, and eating behaviors. Awareness items were scored as 1 = Yes and 0 = No/Do not know, while consumption items were rated on a four-point scale (0–3) based on frequency. Internal consistency of the instrument was confirmed through pilot testing, with Cronbach’s alpha = 0.82 for awareness items and 0.79 for consumption items.

**Results:**

Awareness of junk food hazards was generally high, especially for items such as pizza (91.4%), bakery products (90.1%), and fried chicken (89.9%). Lower awareness was noted for energy drinks (61%) and sweetened fruit drinks (65.9%). Consumption varied significantly by academic year (*p* < 0.001), with Year 2 and Year 3 students reporting the highest intake between meals. Females demonstrated higher awareness (*p* = 0.038) and consumption scores (*p* = 0.012). Regression analysis identified age, gender, self-rated health, and portion size as significant predictors of BMI (*p* < 0.0001).

**Conclusion:**

Despite high awareness, moderate junk food consumption persists, particularly of beverages. Socio-demographic factors such as age, gender, and self-rated health strongly influence dietary habits. Findings highlight the need for targeted campus-based interventions to reduce unhealthy eating among future healthcare professionals.

## 1 Introduction

Eating habits among university students have changed rapidly in recent years, with increasing reliance on convenient, inexpensive, and highly palatable foods. This trend is driven by academic pressures, limited time for meal preparation, aggressive food marketing, and the widespread availability of fast foods across university environments (1–3). Of particular concern is junk food, broadly defined as readily available products high in calories, sugar, fat, and salt, but low in essential nutrients such as fiber, vitamins, and minerals (4, 5). Examples include fried fast foods, confectionery, sweetened beverages, savory snacks, processed meats, and other ultra-processed items (6).

Although junk food is often consumed for its taste, convenience, and social appeal, regular intake is associated with poor dietary quality and long-term health risks (2, 7). University students represent a critical population for studying these behaviors, as dietary patterns established during this formative period often persist into adulthood (8, 9). Health professional students are of particular interest: while they face the same environmental pressures that encourage unhealthy eating, they also represent the future health workforce. Their personal dietary habits may influence their credibility, attitudes toward patient counseling, and willingness to model healthy lifestyle behaviors (10, 11). Understanding junk food awareness and consumption in this group, therefore, has implications not only for their own well-being but also for broader health promotion efforts across university campuses.

In the United Arab Emirates (UAE), rapid economic and nutritional transitions have contributed to increased consumption of energy-dense, nutrient-poor foods among adolescents and young adults (12). This shift has been linked to rising rates of overweight, obesity, and metabolic risk factors, underscoring the growing public health impact of modern dietary patterns (13, 14). While previous studies in the UAE and wider Gulf region have examined general dietary habits, obesity trends, and lifestyle risk factors among university students, limited evidence specifically addresses junk food awareness, consumption patterns, and the socio-demographic determinants of these behaviors among health professional students. Much of the existing research has focused broadly on nutritional status or fast-food intake without evaluating the gap between knowledge of unhealthy food choices and actual consumption, highlighting a significant area for further investigation (15).

Addressing this gap is particularly relevant in the UAE, where lifestyle-related conditions such as obesity, metabolic syndrome, and diabetes continue to rise (16). This study, therefore, investigates the socio-demographic factors influencing junk food consumption among health professional students. As future healthcare providers, their health behaviors may affect both their professional practice and patient counseling. By assessing awareness and consumption patterns, this study aims to identify the factors associated with dietary choices and provide evidence to inform targeted health promotion initiatives within universities.

## 2 Materials and methods

### 2.1 Study design

A quantitative, descriptive cross-sectional study was undertaken to examine the socio-demographic correlates of junk food consumption among undergraduate students enrolled in medical, dental, pharmacy, and nursing programs at a Health Sciences University. Ethical approval was obtained from the Research and Ethics Committee of the institution [Reference: RAKMHSU-REC-213-2022/23-F-N].

### 2.2 Study setting

The study was conducted at a semi-government Health Sciences University located in Ras Al Khaimah, in the northern emirates of the United Arab Emirates. The university hosts diverse undergraduate cohorts across Medicine, Dentistry, Pharmacy, and Nursing programs.

### 2.3 Study population

The study population comprised undergraduate students of both sexes, representing all academic years and programs.

### 2.4 Sample size

The required sample size was calculated using the Raosoft sample size calculator (Raosoft, Inc., 2004) based on a total student population of 1,245, with a 5% margin of error, 95% confidence level, and 50% response distribution. The minimum required sample size was 294. To strengthen validity, all eligible and consenting students present during data collection were included, yielding a final sample of 694 participants.

### 2.5 Data collection instrument

Data were collected using a semi-structured, self-administered questionnaire developed in English, drawing from existing literature on similar research topics (17–19). Content validity was established through expert review, and reliability was tested in a pilot study of 30 students (excluded from the final sample). Cronbach’s alpha values indicated good internal consistency (0.82 for awareness items, 0.79 for consumption frequency items).

The questionnaire comprised of four sections. Section I comprised of socio-demographic data [age, gender, academic year, program of study- Medicine, Dentistry, Pharmacy, and Nursing, height, weight, family and living status, junk food consumption frequency, weekly expenditure in AED, and self-rated health].

Section II included five questions that assessed general awareness of junk food (an example of the item was “Junk foods contain less nutrient and are unhealthy for all age groups”) and ten questions regarding knowledge of health hazards of junk food consumption (an example of the item was “Frequent intake of junk-food leads to extra weight gain and obesity”) using a three-point response format [Yes, No, I do not know]. For every item, a score of 1 was assigned if the participant responded “Yes,” indicating correct awareness. Responses of “No” or “Do not know” were both assigned a score of 0, as they reflected lack of awareness. The total awareness score for each participant was obtained by summing the scores across all items.

Section III evaluated junk food consumption preferences on a four-point scale [Mostly, Often, Sometimes, Never]. The question was “How frequently do you consume the following?”. The items included were fried chicken, pizza, Chinese food, French fries, sausage, bakery items, donuts, chips, ice-cream, chocolates, soft drinks, energy drinks, Fresh fruit juice, sweetened fruit drinks and coffee. The responses were converted into numerical values of 3, 2, 1, and 0, respectively. These item-level scores were summed up to generate a total consumption score, where higher values indicated more frequent intake of junk food items. Both total scores were used as continuous variables for subsequent statistical analyses.

Section IV assessed attitudes toward junk food preferences through eight structured items. There were three questions asked with four response categories “Why do you prefer junk food?” (Delicious taste, Attractive advertisements, Easy availability, Lack of other suitable option); “What time do you prefer to eat junk food?” (11.00 a.m.-12. 00 p.m, 1.00–6.00 p.m., 7.00–10.00 p.m. after 10.00 p.m.); “Where do you get lunch on a typical university day?” (I do not eat my lunch, I go home during lunch time, I eat lunch at campus cafeteria, I eat lunch outside such as restaurant, café) and five questions were asked with three response categories: “How much portion size of junk food do you usually eat?” (Large, Medium, Small); Do you feel your junk food habits is increasing day by day? (Yes, no, I am not sure); Do you eat junk food in between regular meals? (Yes, no, Sometimes); Do you have the habits of checking the nutrient fact label in the junk food? (Yes, no, Sometimes); How many times in a day do you eat the junk food? (Once a day, Two times day, more than two times a day). Portion size categories (small, medium, large) were based on standardized descriptions provided to participants to minimize subjective variation. Students were instructed to classify their usual portion according to the examples given (e.g., “small = minimal amount,” “medium = standard single serving,” “large = more than one serving or a visibly large portion”).

### 2.6 Data collection procedure

Participation was voluntary, with informed consent obtained before questionnaire administration. No incentives were provided. Data were collected face-to-face in classrooms during free periods between September 29 and November 15, 2024. Of 718 responses, 24 incomplete questionnaires were excluded, resulting in 694 valid responses (response rate: 58%).

### 2.7 Data analysis

Data were analyzed using SPSS version 29. Categorical variables were summarized using frequencies and percentages. Continuous variables, such as age and weekly junk food expenditure, were presented as means and standard deviations. Chi-square tests assessed associations between sociodemographic variables and levels of awareness regarding junk food and its health implications. ANOVA was used to compare mean awareness and consumption scores across academic programs and demographic groups. Multiple linear regression analyses examined the impact of predictors such as age, sex, and weekly expenditure on consumption frequency and BMI. Regression coefficients with 95% confidence intervals and *p*-values were reported to identify significant predictors. A p-value of <0.05 was considered statistically significant. This approach allowed for a comprehensive evaluation of junk food consumption patterns and their associations with sociodemographic factors in the study population.

## 3 Results

**Figure 1:**
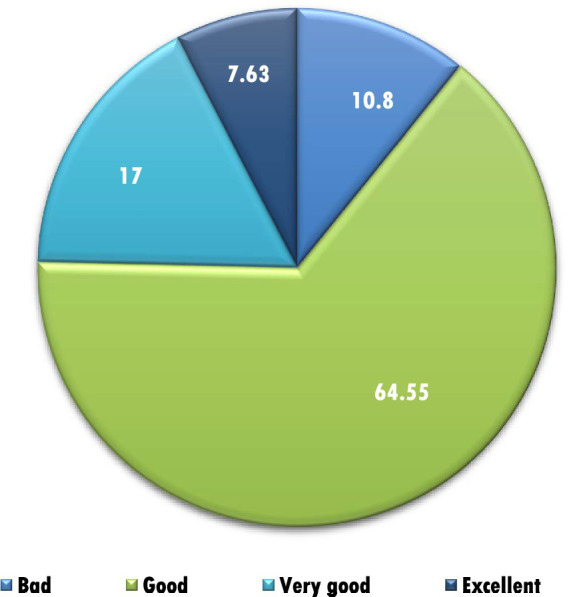
Self-reported health ratings (*n* = 694).

A total of 694 undergraduate students participated (mean age 20.3 ± 1.9 years). The majority were female (72.1%), and most lived with parents in nuclear families. Students were distributed across all academic years, with Year 3 comprising the largest group (34.1%). Weekly expenditure on junk food varied, with one-third of students reporting spending more than 40 AED (Table 1). Most participants rated their health as “good” (64.5%), followed by “very good” (17%) and “excellent” (7.6%) (Figure 1).

**Table 1:** Sociodemographic profile of study participants (*n* = 694).

Sociodemographic variable	Frequency [%]
Age	
< 18 years	118 [17]
19–20	304 [43.8]
21–22	196 [28.2]
23–24	59 [8.5]
>24 years	17 [2.4]
Mean ± SD = 20.27 ± 1.91	
Gender	
Male	194 [27.9]
Female	500 [72.1]
Year of study	
1	144 [20.7]
2	188 [27.1]
3	237 [34.1]
4	55 [7.9]
5	70 [10.2]
Type of family	
Nuclear	423 [60.9]
Joint	271 [39.1]
Living with whom?	
Parents	484 [67.5]
Relative	18 [2.5]
Hostel	123 [17.1]
Friends	14 [2]
Alone	55 [7.9]
Weight in kg [Mean ± SD]	
Boys	79.54 **±** 17.28
Girls	60.21 **±** 12.49
Height in meter [Mean ± SD]	
Boys	1.76 **±** 0.075
Girls	1.61 **±** 0.066
Money spent on junk food	
<10	62
11–20	140
21–30	143
31–40	120
>40	229

**Figure 2:**
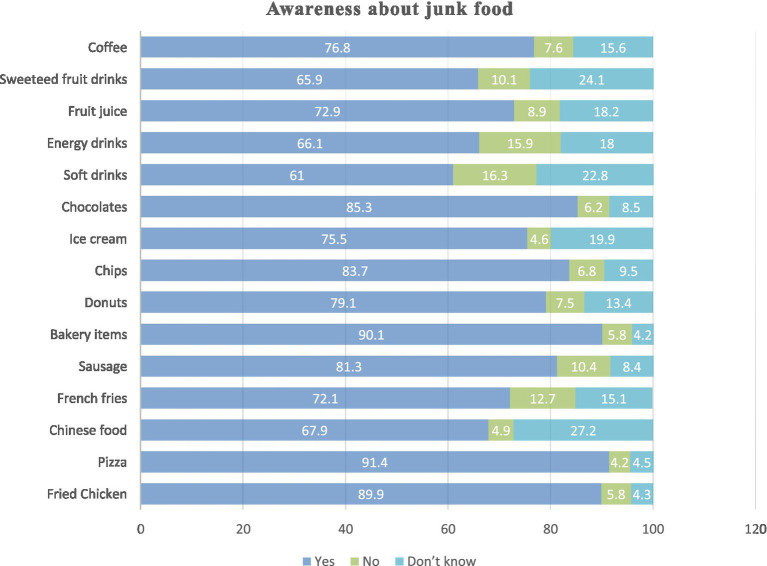
Awareness about junk food among study participants (*n* = 694).

Awareness was generally high for common items such as pizza (91.4%), bakery products (90.1%), fried chicken (89.9%), chips (88.7%), and chocolates (85.3%). Awareness was lower for beverages, including energy drinks (61.0%) and sweetened fruit drinks (65.9%). Items such as sausages, French fries, and Chinese food showed higher proportions of “do not know” responses, reflecting uncertainty among students (Figure 2).

**Figure 3:**
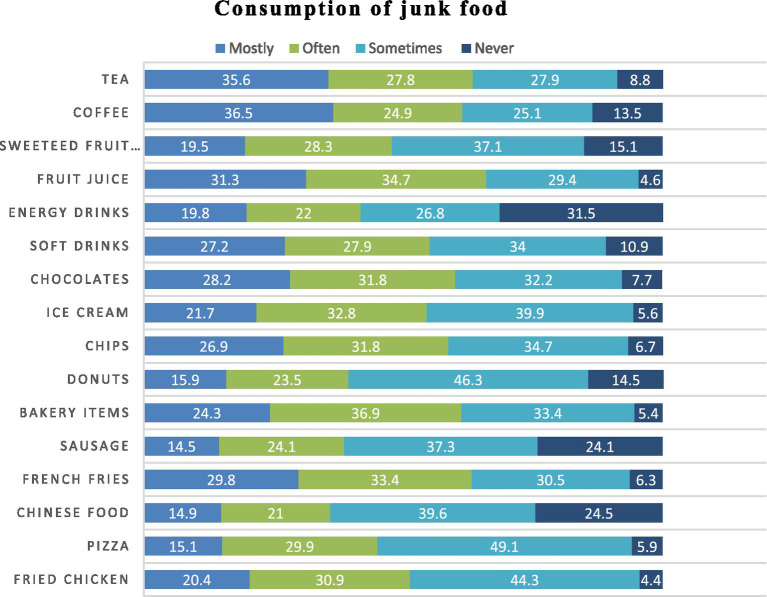
Consumption of junk food among study participants (*n* = 694).

Despite high awareness, moderate consumption persisted. Beverages and snack items were most frequently consumed: tea (63.4% “mostly/often”), coffee (61.4%), soft drinks (55.1%), chocolates (60.0%), and chips (58.7%). Fried chicken and French fries also showed substantial intake. In contrast, energy drinks, donuts, sausages, and Chinese food were consumed less frequently, with higher proportions reporting “sometimes” or “never” (Figure 3).

Table 2 presents the mean awareness and consumption scores for junk food across various socio-demographic groups among the study participants (*n* = 694). Gender-wise comparison showed that females had significantly higher awareness scores than males (11.77 ± 3.51 vs. 11.12 ± 3.74, *p* = 0.038), and also demonstrated significantly higher consumption scores (28.39 ± 10.43 vs. 26.23 ± 9.91, *p* = 0.012). No statistically significant differences in awareness or consumption scores were observed between students from nuclear and joint families. When analyzed by year of study, awareness scores did not differ significantly, although Year 4 students showed the highest mean awareness score. Consumption scores varied significantly across academic years (*p* < 0.001), with Year 2 students reporting the highest consumption levels. Living status showed no significant association with either awareness or consumption scores. Weekly expenditure on junk food was not significantly associated with awareness; however, consumption scores increased progressively with higher spending categories, with a statistically significant difference across groups (*p* = 0.001).

**Table 2:** Mean difference of awareness score and consumption score of junk food according to socio-demographic variables (*n* = 694).

Scores	Gender		t or *f* value	*p* value
	Male	Female		
Mean awareness score ± SD	11.12 ± 3.74	11.77 ± 3.51	2.080	0.038*
Mean Consumption score ± SD	26.23 ± 9.91	28.39 ± 10.43	2.531	0.012*

Scores	Type of family		t or *f* value	*p* value
	Nuclear	Joint		
Mean awareness score ± SD	11.74 ± 3.45	11.34 ± 3.79	1.42	0.15
Mean Consumption score ± SD	27.86 ± 10.97	27.66 ± 9.24	0.25	0.8

Scores	Year of study					t or *f* value	*p* value
	Year 1	Year 2	Year 3	Year 4	Year 5		
Mean awareness score ± SD	11.1 ± 3.2	11.67 ± 3.54	11.53 ± 3.9	12.64 ± 2.9	11.79 ± 3.7	2.03	0.088
Mean Consumption score ± SD	26.44 ± 8.3	31.03 ± 12.1	26.66 ± 9.54	26.49 ± 9.1	26.64 ± 10.7	6.575	<0.001*

Scores	Living status					t or *f* value	*p* value
	Parents	Relative	Hostel	Friends	Alone		
Mean awareness score ± SD	11.55 ± 3.7	9.5 ± 4.5	12.06 ± 2.9	10.93 ± 2.5	11.65 ± 3.9	2.19	0.068
Mean Consumption score ± SD	27.98 ± 10.1	23.83 ± 11.1	28.12 ± 11.3	24.36 ± 5.5	27.45 ± 10.9	1.134	0.339

Scores	Average weekly expense on junk food [AED]					t or *f* value	*p* value
	< 10	11–20	21–30	31–40	Over 40		
Mean awareness score ± SD	11.38 ± 4.1	11.23 ± 3.6	11.85 ± 3.5	11.59 ± 3.4	11.69 ± 3.6	0.634	0.638
Mean Consumption score ± SD	24.62 ± 12.2	26.27 ± 9.5	27.29 ± 10.4	27.75 ± 10.2	29.9 ± 9.9	4.819	0.001*

*Significant *p* < 0.05 level.

Gender-specific differences were noted in portion size (*p* < 0.0001), with males more likely to report large portions, and in eating between meals (*p* < 0.001), where females more often reported “sometimes.” Lunch source also differed (*p* = 0.012), with females more frequently eating at campus cafeterias and males reporting restaurant meals or skipping lunch (Table 3).

**Table 3:** Gender wise difference in junk food preferences among study participants (*n* = 694).

Questions	Responses	Male	Female	χ^2^ value	*p* value
Why do you prefer junk food	Delicious taste	110	312	2.041	0.56
	Attractive ads	14	34		
	Easily available	54	125		
	Lack of other options	15	29		
What time do you prefer junk food?	8 a.m. to 12 p.m.	19	44	4.72	0.193
	1–6 pm	65	197		
	7–10 p.m.	82	212		
	After 10 p.m.	28	47		
How much portion of junk food do you usually eat?	Large	49	49	36.48	<0.0001*
	Medium	118	306		
	Small	27	145		
Do you feel your junk food habits is increasing?	Yes	62	148	3.684	0.159
	No	95	222		
	Not sure	37	130		
Do you eat junk food between regular meals?	Yes	63	109	14.48	<0.001*
	No	97	242		
	Sometimes	34	149		
Do you check nutrient fact labels on junk food?	Yes	75	156	3.685	0.158
	No	75	209		
	Sometimes	44	135		
Where do you get lunch on a typical university day?	No lunch	48	149	10.928	0.012*
	Home	63	120		
	Campus cafeteria	40	146		
	Restaurants	43	85		
How many times in a day do you eat junk food	Once a day	144	379	0.593	0.743
	Two times a day	41	94		
	More than 2 times/day	9	27		

*Significant *p* < 0.05 level.

Year-wise comparisons revealed significant variation in portion size (*p* = 0.0198), perceived increase in junk food habits (*p* = 0.004), and eating between meals (*p* = 0.002). Year 3 students were most likely to consume junk food as snacks and report larger portions. Lunch source also varied, with Year 1 and Year 3 students more often reporting cafeteria meals, while Year 5 students reported restaurant lunches (Table 4).

**Table 4:** Year wise difference in junk food preferences among study participants (*n* = 694).

Questions	Responses	Year 1	Year 2	Year 3	Year 4	Year 5	χ^2^ value	*p* value
Why do you prefer junk food	Delicious taste	99	115	140	32	35	14.81	0.25
	Attractive ads	6	16	20	2	6		
	Easily available	28	50	62	16	22		
	Lack of other options	11	7	15	5	7		
What time do you prefer junk food?	8 a.m. to 12 p.m.	13	19	22	4	5	15.45	0.22
	1–6 pm	44	82	93	19	24		
	7–10 p.m.	62	69	104	26	33		
	After 10 p.m.	25	18	18	6	8		
How much portion of junk food do you usually eat?	Large	10	28	40	4	16	18.19	0.0198*
	Medium	94	123	137	35	35		
	Small	40	37	60	16	19		
Do you feel your junk food habits is increasing?	Yes	29	53	89	16	23	22.39	0.004*
	No	65	91	96	29	36		
	Not sure	50	44	52	10	11		
Do you eat junk food in between regular meals?	Yes	19	49	75	9	20	23.67	0.002*
	No	78	88	106	28	39		
	Sometimes	47	51	56	18	11		
Do you check nutrient fact label on junk food?	Yes	45	61	83	17	25	3.5	0.899
	No	60	82	92	20	30		
	Sometimes	39	45	62	18	15		
Where do you get lunch on a typical university day?	No lunch	37	47	74	14	25	22.51	0.032*
	Home	40	53	57	13	20		
	Campus cafeteria	46	50	63	21	6		
	Restaurants	21	38	43	7	19		
How many times in a day do you eat junk food	Once a day	117	134	173	41	58	9.549	0.298
	Two times a day	22	44	50	9	10		
	More than 2 times/day	5	10	14	5	2		

*Significant *p* < 0.05 level.

Multiple linear regression was conducted to identify predictors of body mass index (BMI) among participants. Age, gender, self-rated health, and portion size of junk food emerged as significant predictors (Table 5). Specifically, BMI increased with age (B = 0.322, *p* < 0.0001) and larger portion sizes (B = 0.909, *p* < 0.0001). In contrast, female gender (B = −2.074, *p* < 0.0001) and better self-rated health (B = −0.793, *p* < 0.0001) were associated with lower BMI. These findings suggest that both demographic and behavioral factors contribute meaningfully to variations in BMI among health sciences students.

**Table 5:** Multiple linear regression model depicting significant predictor variables for BMI among study participants (*n* = 694).

Variable	Regression coefficient	Wald’s statistic [t]	*p*-value	95% confidence interval	
				Lower bound	Upper bound
Constant	22.47	11.35	<0.0001*	18.58	26.536
Age (years)	0.322	3.72	<0.0001*	0.15	0.49
Gender (male vs female)	−2.074	−5.51	<0.0001*	−2.81	−1.33
Rating own health (self-rated health)	−0.793	−3.55	<0.0001*	−1.23	−0.35
Portion of junk food (portion size/unit)	0.909	3.31	<0.0001*	0.37	1.45

*Significant *p* < 0.05 level.

## 4 Discussion

This study sheds light on socio-demographic factors influencing junk food consumption among health professional students in the United Arab Emirates. Although awareness of the health risks associated with junk food was high, moderate levels of intake persisted, particularly in the form of beverages such as tea, coffee, and soft drinks. This disconnect between knowledge and practice highlights the broader challenge of converting awareness into healthier eating behaviors. Gender differences were notable: female students reported both greater awareness and higher consumption scores. Comparable findings have been observed in other UAE university cohorts, where stress, academic demands, and lifestyle pressures contributed to unhealthy eating habits (20). These results suggest that interventions should be tailored to gender-specific needs, addressing both social influences and environmental constraints. Patterns related to year of study and spending also emerged as significant. Students in their second and third years reported higher consumption, likely reflecting heavier academic workloads, stress, and reliance on inexpensive, quick meals. Unsurprisingly, students who allocated more money to junk food consumed it more frequently, underscoring the role of affordability and accessibility. The increasing use of online food delivery platforms has further normalized junk food consumption, with convenience and cost acting as major drivers (21).

Regression analysis revealed that age, gender, self-rated health, and portion size were significant predictors of BMI. Students who rated their health more positively tended to have lower BMI, suggesting that self-perception may align with healthier dietary practices. Comparable studies reinforce these findings: Algamdi (22) reported that gender and self-esteem strongly influenced BMI among Saudi university students. Rojo-Ramos et al. (23) found similar gender-based differences in body image and BMI among Spanish cohorts. Wang et al. (24) demonstrated that higher BMI was associated with poorer self-rated health in Chinese adults and Joshi et al. (25) highlighted that Indian adolescents with higher BMI reported lower self-esteem.

Within the UAE, Al Sabbah et al. and earlier studies in Ajman and Dubai confirmed that dietary behaviors, reliance on food delivery, and lifestyle factors significantly shaped BMI outcomes among female students (26–28). Together, these studies validate that age, gender, and self-perception are consistent predictors of BMI across diverse contexts, underscoring the importance of integrating behavioral counseling and self-awareness into health promotion strategies. Placing these results within the UAE’s public health context is essential. Non-communicable diseases account for 55% of deaths nationally and impose an economic burden of nearly AED 40 billion annually, equivalent to 2.7% of GDP (29). Dietary risks are a major contributor, and health professional students as future providers represent a critical group whose personal habits may influence both their own health and their ability to counsel patients. Addressing junk food consumption among this population is therefore both a matter of individual well-being and professional responsibility. The Ministry of Health and Prevention’s 2023–2026 strategy and the 2024 National Plan to Combat Noncommunicable Diseases emphasize promoting healthier lifestyles and reducing diet-related risks (30, 31). Overall, the findings highlight that awareness alone is insufficient. Structural measures — such as improving access to affordable healthy food options on campuses, embedding nutrition education into curricula, and designing culturally sensitive campaigns—are necessary to shift behaviors. By situating these findings within the UAE’s socio-economic and cultural context, this study provides evidence to inform both policy and practice aimed at reducing the burden of diet-related NCDs.

### 4.1 Limitations

In the present study, which has a cross-sectional design, inferences on the causal links between sociodemographic factors and junk food consumption cannot be assured. A longitudinal design is thus desirable to track temporal changes in eating behavior and their influence on health outcome measures. Second, self-reported measures may lead to bias owing to social desirability. Also, although the questionnaire included items assessing general awareness of junk food and a set of ten questions related to health hazards, it did not comprehensively evaluate students’ detailed understanding of the specific harmful health effects associated with the consumption of individual junk food products. As such, while overall awareness was captured, the tool may not fully reflect the depth or breadth of participants’ knowledge regarding the medical and physiological consequences of junk food intake. This limitation may have led to a partial underestimation of true knowledge levels.

## 5 Conclusion

This study provides clear evidence that while health professional students in the UAE demonstrate high levels of awareness regarding junk food and its health risks, the awareness is not necessarily converted into healthier eating habits. Socio-demographic differences were notable: females demonstrated higher awareness and consumption scores, while students in their second and third academic years showed significantly higher consumption, especially between meals. Consumption patterns were highly correlated with weekly expenditure, emphasizing the role of affordability and accessibility. More interestingly, the regression model revealed that age, gender, self-rated health, and portion size were the significant predictors for BMI, indicating the cumulative effects of demographic and behavioral factors on the nutritional status of students.

These findings have several practical implications for university health promotion strategies. The interventions need to be advanced beyond knowledge-based approaches by including behavioral, environmental, and structural elements, such as increasing the healthy food availability on campus, banning aggressive marketing of unhealthful foods, and incorporating nutrition counselling into the health sciences curriculum. From a policy perspective, universities can clearly support the UAE’s national NCD prevention agenda by implementing campus-wide wellness policies that include portion control and promote culturally tailored dietary advice.

For future research, longitudinal studies are needed to clarify causal pathways between awareness, behavior, and BMI, as well as to evaluate targeted interventions that address gender-specific needs, spending habits, and stress-related eating patterns. Qualitative studies may also provide an explanation of why knowledge does not always translate into healthier choices. Overall, this study emphasizes the need for multi-level, evidence-based interventions aimed at reducing junk food consumption and promoting healthier lifestyles among prospective health professionals.

## Data Availability

The raw data supporting the conclusions of this article will be made available by the corresponding author upon request.
